# Dorsomorphin attenuates ABCG2-mediated multidrug resistance in colorectal cancer

**DOI:** 10.3389/fphar.2024.1393693

**Published:** 2024-05-24

**Authors:** Xiao-Peng Li, Liang-Qi Cao, Ze-Zhong Yu, Ke He, Peng-Bo Ding, Ji-Sheng Li, Yi-Yao Shan, Yu-Bin Su, Zhong-Min Yuan, Zhi Shi

**Affiliations:** ^1^ Cancer Minimally Invasive Therapies Centre, Guangdong Second Provincial General Hospital, Jinan University, Guangzhou, China; ^2^ Department of Cell Biology and Institute of Biomedicine, Guangdong Provincial Biotechnology and Engineering Technology Research Center, Guangdong Provincial Key Laboratory of Bioengineering Medicine, Genomic Medicine Engineering Research Center of Ministry of Education, MOE Key Laboratory of Tumor Molecular Biology, National Engineering Research Center of Genetic Medicine, State Key Laboratory of Bioactive Molecules and Druggability Assessment, College of Life Science and Technology, Jinan University, Guangzhou, China; ^3^ Key Laboratory of Neurogenetics and Channelopathies of Guangdong Province and the Ministry of Education of China, The Second Affiliated Hospital, Institute of Neuroscience, Guangzhou Medical University, Guangzhou, China; ^4^ Department of Hepatobiliary Surgery, The Second Affiliated Hospital of Guangzhou Medical University, Guangzhou, China

**Keywords:** dorsomorphin, chemosensitivity, ABCG2, multidrug resistance, colorectal cancer

## Abstract

Colorectal cancer is a common malignant tumor with high mortality, for which chemotherapy resistance is one of the main reasons. The high expression of ABCG2 in the cancer cells and expulsion of anticancer drugs directly cause multidrug resistance (MDR). Therefore, the development of new ABCG2 inhibitors that block the active causes of MDR may provide a strategy for the treatment of colorectal cancer. In this study, we find that dorsomorphin (also known as compound C or BML-275) potently inhibits the transporter activity of ABCG2, thereby preserving the chemotherapeutic agents mitoxantrone and doxorubicin to antagonize MDR in ABCG2-overexpressing colorectal cancer cells. Additionally, dorsomorphin does not alter ABCG2 protein expression. The results of molecular docking studies show that dorsomorphin is bound stably to the ABCG2-binding pocket, suggesting that dorsomorphin is a potent ABCG2 inhibitor that attenuates ABCG2-mediated MDR in colorectal cancer.

## 1 Introduction

Cancer multidrug resistance (MDR) refers to the resistance of cancer cells to various anticancer agents that are unrelated to structure and function, which reduces the effects of chemotherapy and is not conducive to the survival of cancer patients (Fan et al., 2023). An important reason for the development of MDR is ABCG2 overexpression in the cancer cells, which can excrete the anticancer drugs against the concentration gradient (Li et al., 2016). As one of the ATP-binding cassette transporters, ABCG2 is a transmembrane protein on the cell membrane that can obtain energy to expel substrates out of the cell through ATP hydrolysis (Eckenstaler and Benndorf, 2020). ABCG2 directly supports many anticancer drugs, such as mitoxantrone (Sugimoto et al., 2003), doxorubicin (Stacy et al., 2013), irinotecan (Nielsen et al., 2017), imatinib (Noguchi et al., 2009), dasatinib (Eadie et al., 2014), and erlotinib (Shi et al., 2007). Given the important role of ABCG2 in mediating MDR, effective ABCG2 inhibitors can help reverse MDR. Several research groups have discovered a series of ABCG2 inhibitors, such as AZ32 (Liu et al., 2021), AZ-628 (Wang J. Q. et al., 2020), febuxostat (Miyata et al., 2016), fumitremorgin C (Toyoda et al., 2019), GSK2606414 (Yu et al., 2023), KU55933 (Liu et al., 2022), MK-2206 (Gao et al., 2023), NVP-TAE684 (Wang J. et al., 2020), OTS964 (Yang et al., 2021), and VKIN-1 (Narayanan et al., 2021). However, there is no ABCG2 inhibitor that has been used successfully in clinical settings to reverse cancer MDR. Therefore, there is an urgent need to develop novel ABCG2 inhibitors.

In this study, we investigate the effects of dorsomorphin (also known as compound C or BML-275) on ABCG2 activity and ABCG2-mediated MDR in colorectal cancer. Colorectal cancer is one of the main types of fatal cancers (Ghasemian et al., 2023), and ABCG2 expression has been associated with tumor responses to irinotecan-based or FOLFOX therapy in metastatic colorectal cancer patients (Lin et al., 2013; Palshof et al., 2020). Our findings demonstrate that dorsomorphin is a potent ABCG2 inhibitor that attenuates ABCG2-mediated MDR in colorectal cancer.

## 2 Materials and methods

### 2.1 Reagents and cell culture

Dorsomorphin (#1219188-18-9), KU55933 (#587871-26-9), mitoxantrone (#70476-82-3), doxorubicin (#A603456-0025), cisplatin (#AA1A8019B), rhodamine 123 (#62669-70-9), and 3-(4,5-dimethylthiazol-yl)-2,5-diphenyl-tetrazolium bromide (MTT; #298-93-1) were procured from MREDA Technology Inc. (Beijing, China), TargetMol Chemicals Inc. (Shanghai, China), D&B Biotech Inc. (Shanghai, China), Sangon Biotech Inc. (Shanghai, China), Qilu Pharmaceutical Co. (Jinan, China), Sigma-Aldrich Trading Co. (Shanghai, China), and Yuanye Biotech Co. (Shanghai, China), respectively. Anti-ABCG2 antibody (#RLT0053) and anti-β-actin antibody (#SC-47778) were purchased from Ruiying Biotech (Wuxi, China) and Santa Cruz Biotech (Santa Cruz, California, United States), respectively. Human colorectal cancer cells S1-M1-80 vector with ABCG2 overexpression and S1-M1-80 sgABCG2 with ABCG2-knockout were established as reported previously (Liu et al., 2021) and cultured in Dulbecco’s modified Eagle’s medium (#C11995500BT) with 10% fetal bovine serum (#10270-106) from Thermo Fisher Scientific Inc. (Waltham, Massachusetts, United States) at 37°C in a humid atmosphere of 5% CO_2_.

### 2.2 Cytotoxicity assay

The cells were cultured in 96-well plates at 7 × 10^3^ cells/well and treated with the indicated agents for 72 h. After incubating with 500 mg/mL MTT for another 4 h and discarding the solution in the wells, approximately 50 μL of dimethylsulfoxide (DMSO) was added to each well. The absorbance was then detected at 570 nm with a BioTek Synergy H1 microplate reader from Agilent Technologies Inc. (Santa Clara, California, United States). The Bliss method was used to calculate the 50% inhibitive concentration (IC_50_), as reported previously (Zhang et al., 2017).

### 2.3 Drug accumulation assay

The cells were cultured in 12-well plates at 5 × 10^4^ cells/well and incubated with the indicated concentration of dorsomorphin or KU55933 for 1 h. After incubating with mitoxantrone, doxorubicin, or rhodamine 123 at a concentration of 10 μM for another 2 h, the images of the cells were acquired using the LSM900 confocal microscope from Carl Zeiss Inc. (Oberkohen, Germany). Next, the cells were collected and analyzed with a CytoFLEX flow cytometer from Beckman Coulter Inc. (Brea, California, United States), as reported previously (Liu et al., 2022).

### 2.4 Western blot

The cells were lysed with a lysis buffer (containing 1% NP-40, 0.5% sodium deoxycholate, 0.1% SDS, 10 ng/mL phenylmethanesulfonyl fluoride, 0.03% aprotinin, and 1 µM sodium orthovanadate) at 4°C for 30 min. After centrifuging for 10 min at 1.4 × 10^4^ *g*, the protein supernatants were collected and separated using 10% SDS-PAGE gels and transferred to polyvinylidene difluoride membranes. After blocking with 5% bovine serum albumin, the membranes were incubated with the specified primary antibodies and the corresponding horseradish-peroxidase-conjugated secondary antibodies. The signals were then acquired and examined using a ChemiDoc XRS chemiluminescent gel imaging system from Analytik Jena AG (Thuringia, Germany).

### 2.5 Docking analysis

The human ABCG2 protein crystal structure was archived from the Protein Data Bank (PDB ID: 6vxi). The molecular dockings of dorsomorphin and ABCG2 were analyzed using AutoDock Vina, and the data were visualized using PyMOL.

### 2.6 Statistical analysis

The significant differences were determined using Student’s t-test in GraphPad prism 8.3.0, and a *p*-value <0.05 was considered to be statistically significant.

## 3 Results

### 3.1 Dorsomorphin restores the chemosensitivity of colorectal cancer cells with ABCG2 overexpression

**FIGURE 1:**
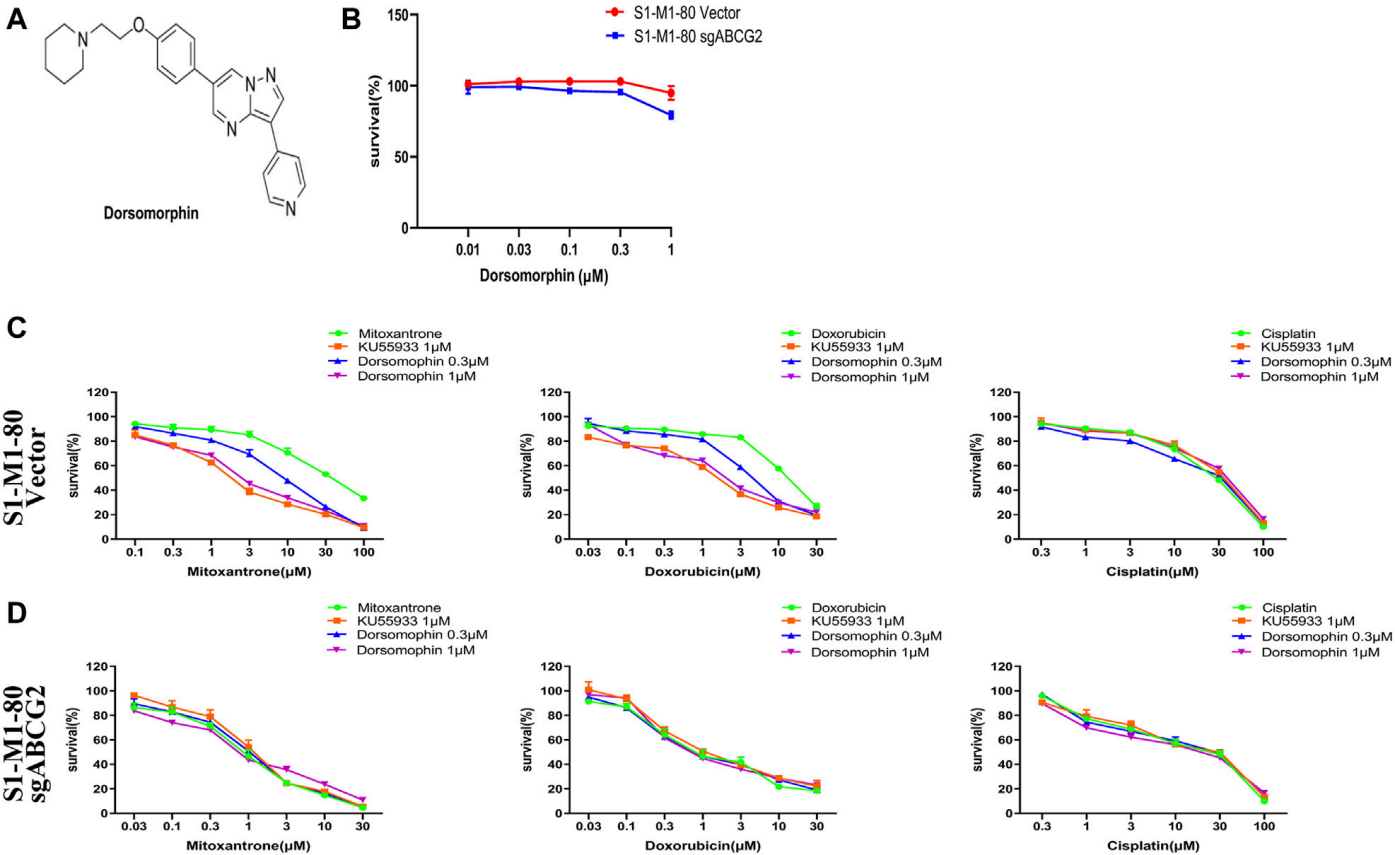
Dorsomorphin restores the chemosensitivity of colorectal cancer cells with ABCG2 overexpression. **(A)** Chemical structure of dorsomorphin. The cells were treated with the indicated agents for 72 h and examined via MTT assay. The representative cell survival curves are shown in **(B–D)**.

To investigate the effects of dorsomorphin (whose chemical structure is shown in Figure 1A) on colorectal cancer cells with ABCG2 overexpression, we first performed the MTT assay to assess the cytotoxicity of dorsomorphin in both the S1-M1-80 vector and S1-M1-80 sgABCG2 cells. Dorsomorphin at 1 μM did not show cytotoxicity in both types of cells (Figure 1B). Therefore, dorsomorphin was applied at concentrations of 0.3 μM and 1 μM to examine its sensitizing effects. As presented in Figures 1C, D and Table 1, compared with the known ABCG2 inhibitor KU55933, dorsomorphin relatively restores the chemosensitivity of the ABCG2 substrate mitoxantrone and doxorubicin in a dose-dependent manner only in the S1-M1-80 vector cells and not in the S1-M1-80 sgABCG2 cells. Both KU55933 and dorsomorphin are unable to restore the chemosensitivity of the non-ABCG2 substrate cisplatin in both types of cells. These results suggest that dorsomorphin can restore the chemosensitivity of colorectal cancer cells with ABCG2 overexpression.

**TABLE 1:** Summary of the IC_50_ values. The fold-reversal value was computed by dividing the IC_50_ of each drug in the S1-M1-80 vector or S1-M1-80 sgABCG2 cells in the absence and presence of inhibitors. *p < 0.05 and **p < 0.01 compared with the corresponding group.

	IC_50_ (μM) ± SD (fold reversal)	
Compound	S1-M1-80 vector	S1-M1-80 sgABCG2
**Mitoxantrone**	31.407 ± 2.119 (1.00)	0.911 ± 0.087 (34.47)
+KU55933 1 μM	2.012 ± 0.067 (15.61)**	1.106 ± 0.113 (30.91)
+Dorsomorphin 0.3 μM	7.180 ± 0.778 (4.37)**	0.849 ± 0.116 (37.01)
+Dorsomorphin 1 μM	2.870 ± 0.243 (10.94)**	0.882 ± 0.177 (35.60)
**Doxorubicin**	11.920 ± 0.605 (1.00)	0.776 ± 0.128 (15.37)
+KU55933 1 μM	1.562 ± 0.069 (7.63)**	0.919 ± 0.084 (12.97)
+Dorsomorphin 0.3 μM	4.783 ± 0.352 (2.49)*	0.749 ± 0.098 (15.91)
+Dorsomorphin 1 μM	2.063 ± 0.194 (5.78)**	0.667 ± 0.049 (17.87)
**Cisplatin**	28.933 ± 0.472 (1.00)	27.830 ± 1.682 (1.04)
+KU55933 1 μM	28.813 ± 1.259 (1.00)	28.000 ± 0.616 (1.03)
+Dorsomorphin 0.3 μM	28.093 ± 1.715 (1.03)	25.820 ± 2.586 (1.12)
+Dorsomorphin 1 μM	27.797 ± 4.721 (1.04)	23.943 ± 0.777 (1.21)

### 3.2 Dorsomorphin increases the ABCG2 substrate levels in colorectal cancer cells with ABCG2 overexpression

**FIGURE 2:**
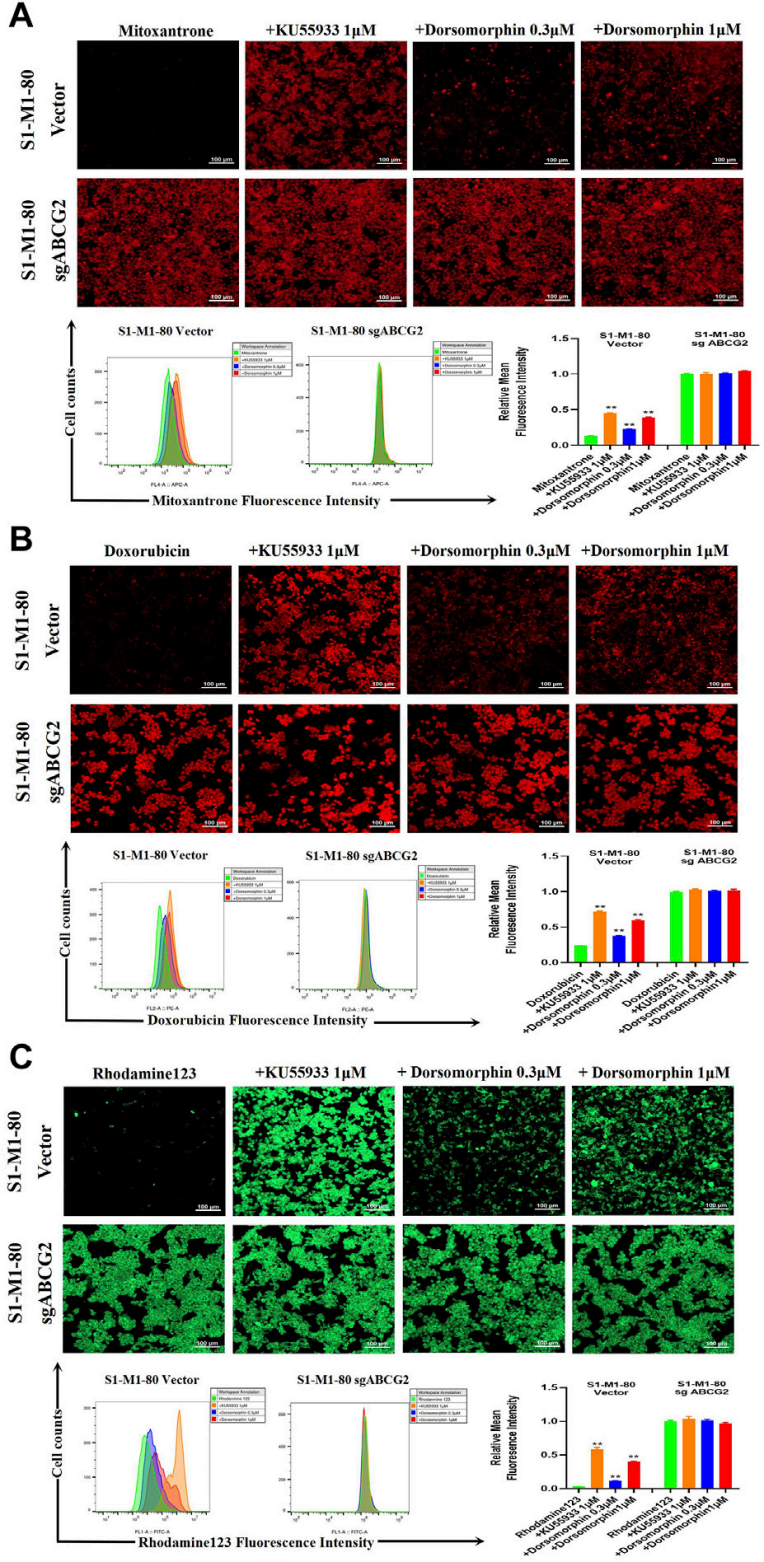
Dorsomorphin increases the ABCG2 substrate levels in colorectal cancer cells with ABCG2 overexpression. The cells were incubated with 10 μM **(A)** mitoxantrone, **(B)** doxorubicin, and **(C)** rhodamine 123 for 2 h after preincubation with dorsomorphin or KU55933 for 1 h and imaged with a confocal microscope, followed by quantification with a flow cytometer. **p < 0.01 compared with the corresponding group.

To further explore whether dorsomorphin could directly suppress the transporter activity of ABCG2, we conducted drug accumulation experiments on the ABCG2 substrates mitoxantrone, doxorubicin, and rhodamine 123 with both the S1-M1-80 vector and S1-M1-80 sgABCG2 cells. As presented in Figure 2A–C, the levels of mitoxantrone, doxorubicin, and rhodamine 123 in the S1-M1-80 sgABCG2 cells are higher than those in the S1-M1-80 vector cells. Moreover, compared with KU55933, dorsomorphin relatively increases the levels of mitoxantrone, doxorubicin, and rhodamine 123 in a dose-dependent manner only in the S1-M1-80 vector cells and not in the S1-M1-80 sgABCG2 cells. These data indicate that dorsomorphin can increase the ABCG2 substrate levels in colorectal cancer cells with ABCG2 overexpression.

### 3.3 Dorsomorphin does not alter the protein level of ABCG2 or the mode of binding with ABCG2

**FIGURE 3:**
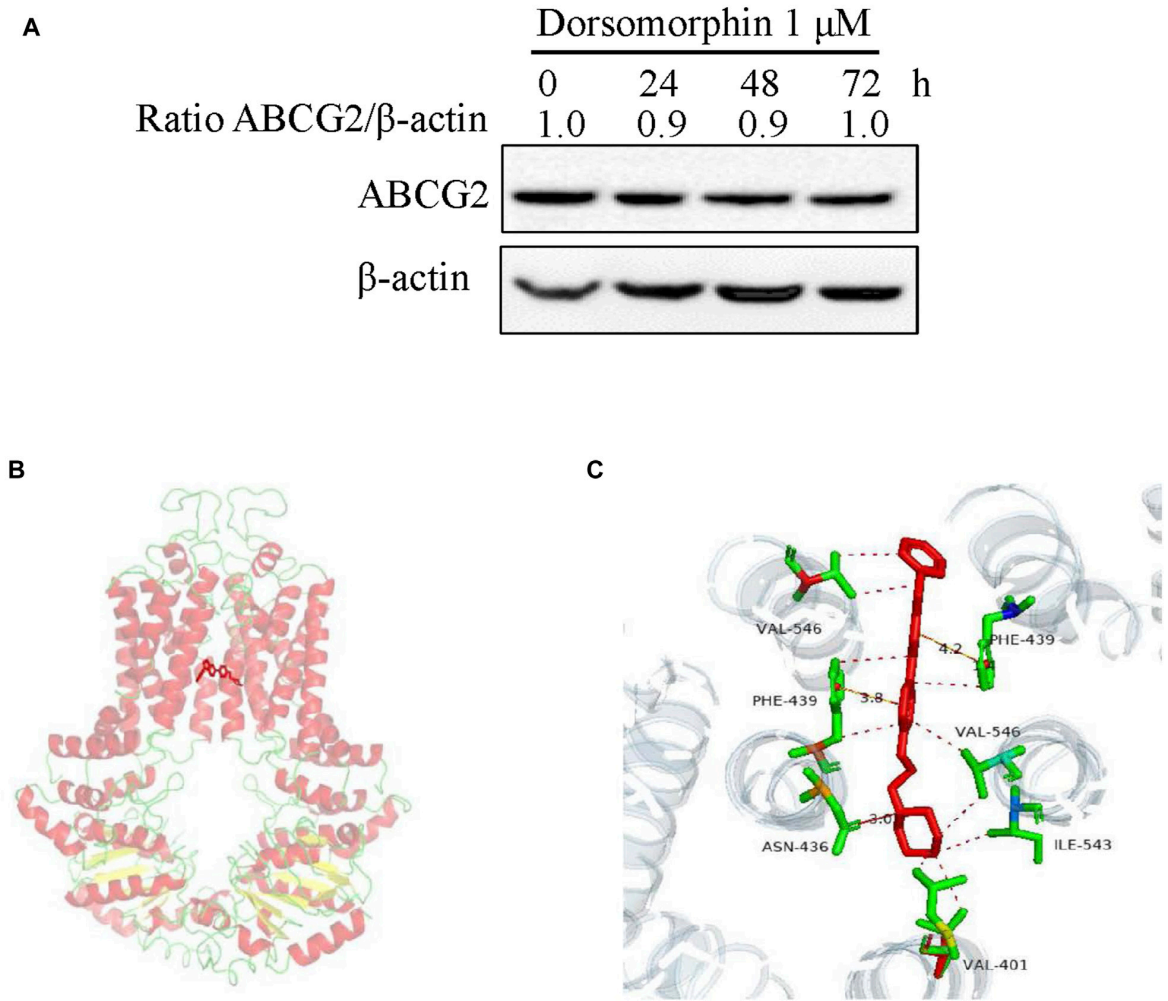
Dorsomorphin does not alter the protein level of ABCG2 or mode of binding with ABCG2. **(A)** ABCG2 expression levels in S1-M1-80 vector cells treated with 1 μM dorsomorphin for the indicated time points were measured via Western blot assay. **(B)** Optimal docked positions of dorsomorphin (red lines) within the binding pocket of human ABCG2 generated using AutoDock Vina. **(C)** Magnification of the highlighted area showing GSK2606414 interactions with the residues Val-401, Asn-436, Phe-439, Ile-543, and Val-546 of ABCG2.

To test the effects of dorsomorphin on the protein level of ABCG2, we treated the S1-M1-80 vector cells with 1 μM dorsomorphin for 24, 48, and 72 h. The results of Western blotting show that dorsomorphin has no effect on the protein level of ABCG2 in the S1-M1-80 vector cells **(** Figure 3A). Next, we carried out a structure-based docking analysis to explore the binding of dorsomorphin with ABCG2. As shown in Figures 3B, C, dorsomorphin is located in the ABCG2-binding pocket, and the hydrophobic amino acid sites, such as Val-401, Asn-436, Phe-439, Ile-543, and Val-546, on ABCG2 engage in hydrophobic interactions to stabilize the binding conformation of dorsomorphin. Dorsomorphin is also observed to form intermolecular hydrogen bonds with Asn-436 of TM2 on ABCG2 and π-π bonds with Ph-439 of TM2 on ABCG2 to further stabilize its binding conformation.

## 4 Discussion

Dorsomorphin, initially named as compound C, was earlier identified as an AMP-activated protein kinase (AMPK; Ki = 109 nM) inhibitor but did not obviously suppress the activities of structurally related kinases, including JAK3, PKA, PKCθ, SYK, and ZAPK (Zhou et al., 2001). Dorsomorphin was also observed to inhibit the bone morphogenetic protein (BMP) type I receptors activin receptor-like kinase (ALK) 2, ALK3, and ALK6, thereby suppressing BMP-induced SMAD1/5/8 phosphorylation and transcription of the target genes in zebrafish (Yu et al., 2008). This compound was named dorsomorphin owing to its ability to induce dorsoventral patterning defects that usually occur in BMP-pathway-mutant zebrafish embryos (Yu et al., 2008). In a profiling study of dorsomorphin against 70 human kinases, dorsomorphin at 1 μM suppressed the activities of 10 out of 70 kinases more powerfully than it suppressed 73% of the AMPK activity, including (from the strongest to weakest) MELK1, PHK, DYRK3, ERK8, DYRK1A, MNK1, Lck, DYRK2, Src, and HIPK2 (Bain et al., 2007). In another profiling study of dorsomorphin against 123 human kinases, dorsomorphin at 1 μM suppressed the activities of 31 out of 123 kinases more powerfully than it suppressed 50% of the AMPK activity, including (from the strongest to weakest) VEGFR, RIPK2, ERK8, GCK, CLK2, DYRK1A, PHK, ABL, CAMKKβ, CK1, NUAK1, MELK, PRK2, YES1, Lck, EPHB2, IRAK4, TrkA, HIPK2, MINK1, IRR, EPHB4, Src, EPHA2, MLK3, FGFR1, DYRK3, EPHB3, CK2, ALK3, and MARK3 (Vogt et al., 2011). In the present study, we found that dorsomorphin at 0.3 μM can inhibit the ABCG2 transporter activity, thereby preserving the chemotherapeutic agents mitoxantrone and doxorubicin to antagonize MDR in ABCG2-overexpressing colorectal cancer cells. Additionally, dorsomorphin does not alter ABCG2 protein expression. The results of molecular docking show that dorsomorphin is bound stably to the ABCG2-binding pockets; therefore, dorsomorphin is a multitarget agent.

The anticancer effects of dorsomorphin have been explored extensively. Dorsomorphin causes cell-cycle arrest at the G2/M phase and apoptosis in glioma cells through AMPK-dependent and -independent mechanisms (Vucicevic et al., 2009). Another study has shown that the dorsomorphin AMPK-independent mode induces G2/M cell-cycle arrest, autophagy, and necroptosis through activation of the calpain/cathepsin pathway and inhibition of AKT/mTORC1/C2 in glioma cells (Liu et al., 2014). Additionally, the dorsomorphin AMPK-independent mode induces apoptosis through increased ceramide production in breast cancer cells (Jin et al., 2009). Dorsomorphin also causes G2/M cell-cycle arrest and growth inhibition by inducing autophagy and apoptosis in human colorectal cancer cells (Yang et al., 2012). Moreover, dorsomorphin inhibits cell growth and migration by interfering with the Akt/mTOR/Wnt pathways in colon cancer cells (Ghanaatgar-Kasbi et al., 2019). The dorsomorphin AMPK-independent mode enhances TRAIL-induced apoptosis through reactive-oxygen-species-mediated decreases of c-FLIPL and Mcl-1 in human renal cancer cells (Jang et al., 2010). Dorsomorphin also AMPK-independently causes cancer cell apoptosis and enhances the sensitivity of cancer cells to both HSP90 and proteasome inhibitors through downregulation of the nuclear heat shock factor 1 (Li et al., 2019). Dorsomorphin sensitizes multiple acute leukemia cells to BH3 mimetic-induced cell death by decreasing the phosphorylation of BAD at Ser75 and Ser99 to enhance BAD translocation to the mitochondria and inhibit BCLX_L_ (Jia et al., 2024). In the present work, our data show that dorsomorphin can restore the sensitivity of mitoxantrone and doxorubicin in colorectal cancer cells with ABCG2 overexpression *in vitro*. However, the effects of dorsomorphin *in vivo* need to be explored in the future. In conclusion, dorsomorphin is a potent ABCG2 inhibitor that can attenuate ABCG2-mediated MDR in colorectal cancer.

## Data Availability

The original contributions presented in the study are included in the article/supplementary material; further inquiries can be directed to the corresponding author.
