# Supplementary figures and images for: Dorsomorphin attenuates ABCG2-mediated multidrug resistance in colorectal cancer
Source: Front Pharmacol. 2024 May 24;15:1393693. doi: 10.3389/fphar.2024.1393693 (PMC11157230; doi:10.3389/fphar.2024.1393693)

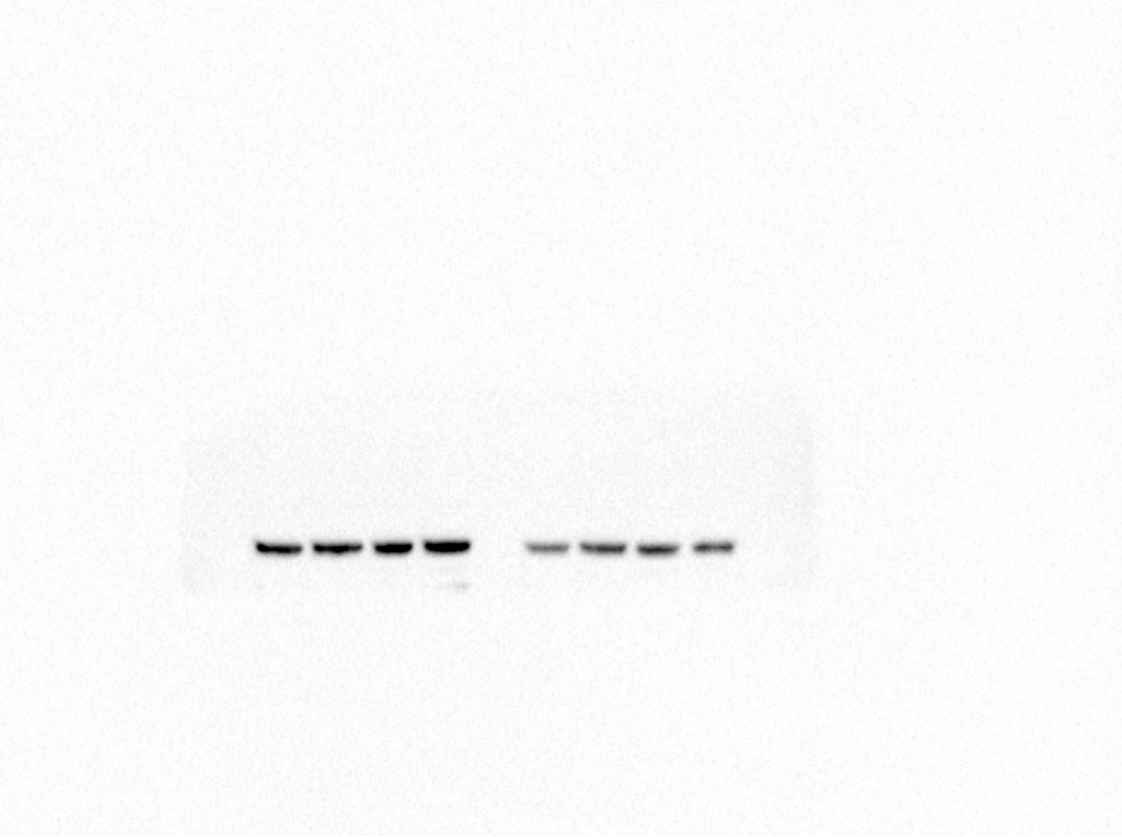

Supplement: Supplementary file 1 [file DataSheet3.ZIP › Original western blot images/ABCG2 in Figure 3 is right side..tif]

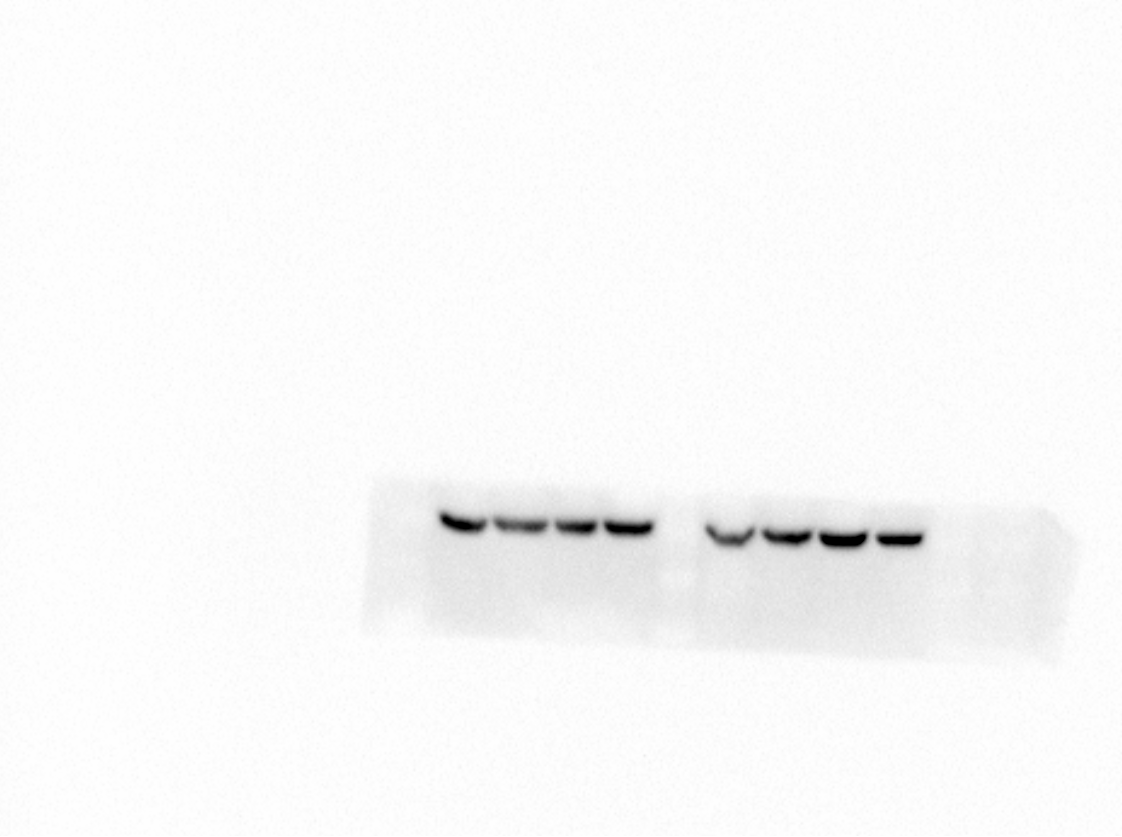

Supplement: Supplementary file 1 [file DataSheet3.ZIP › Original western blot images/Beta-actin in Figure 3 is right side..tif]

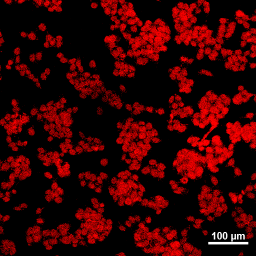

Supplement: Supplementary file 2 [file DataSheet1.ZIP › Raw Data 1/Figure 2/Drug accumulation in fluorescence microscopy/Doxorubicin +Dorsomorphin 0.3μM S1-M1-80 sgABCG2.png]

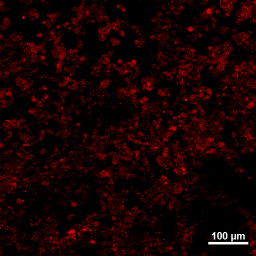

Supplement: Supplementary file 2 [file DataSheet1.ZIP › Raw Data 1/Figure 2/Drug accumulation in fluorescence microscopy/Doxorubicin +Dorsomorphin 0.3μM S1-M1-80 Vector.png]

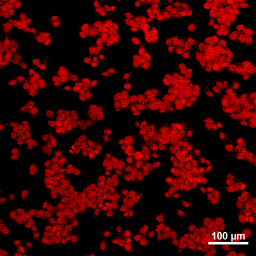

Supplement: Supplementary file 2 [file DataSheet1.ZIP › Raw Data 1/Figure 2/Drug accumulation in fluorescence microscopy/Doxorubicin +Dorsomorphin 1μM S1-M1-80 sgABCG2.png]

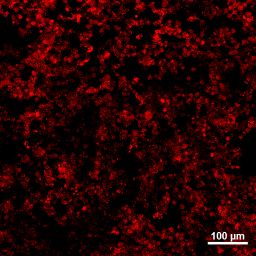

Supplement: Supplementary file 2 [file DataSheet1.ZIP › Raw Data 1/Figure 2/Drug accumulation in fluorescence microscopy/Doxorubicin +Dorsomorphin 1μM S1-M1-80 Vector.png]

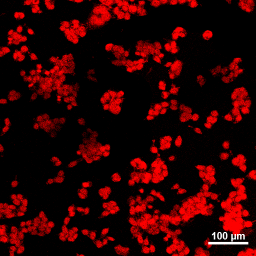

Supplement: Supplementary file 2 [file DataSheet1.ZIP › Raw Data 1/Figure 2/Drug accumulation in fluorescence microscopy/Doxorubicin +KU55933 1μM S1-M1-80 sgABCG2.png]

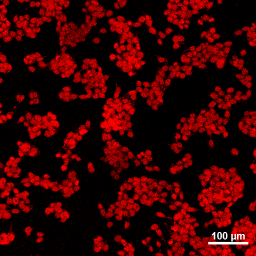

Supplement: Supplementary file 2 [file DataSheet1.ZIP › Raw Data 1/Figure 2/Drug accumulation in fluorescence microscopy/Doxorubicin S1-M1-80 sgABCG2.png]

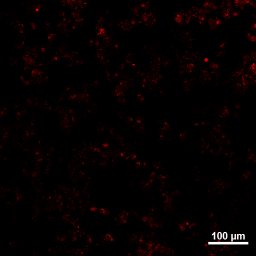

Supplement: Supplementary file 2 [file DataSheet1.ZIP › Raw Data 1/Figure 2/Drug accumulation in fluorescence microscopy/Doxorubicin S1-M1-80 Vector.png]

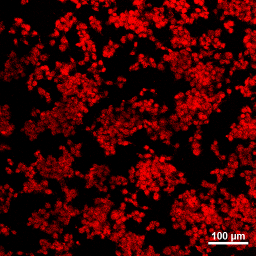

Supplement: Supplementary file 2 [file DataSheet1.ZIP › Raw Data 1/Figure 2/Drug accumulation in fluorescence microscopy/Doxorubicin+KU55933 1μM S1-M1-80 Vector.png]

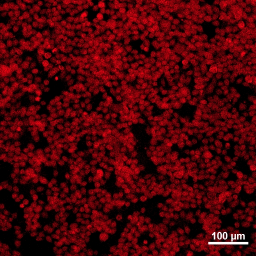

Supplement: Supplementary file 2 [file DataSheet1.ZIP › Raw Data 1/Figure 2/Drug accumulation in fluorescence microscopy/Mitoxantrone +Dorsomorphin 0.3μM S1-M1-80 sgABCG2.png]

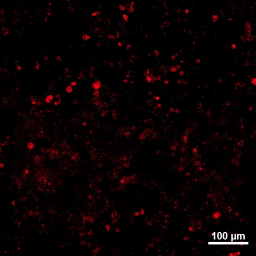

Supplement: Supplementary file 2 [file DataSheet1.ZIP › Raw Data 1/Figure 2/Drug accumulation in fluorescence microscopy/Mitoxantrone +Dorsomorphin 0.3μM S1-M1-80 Vector.png]

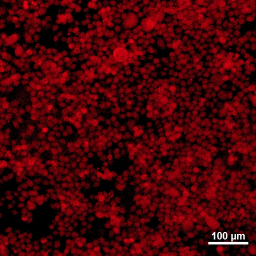

Supplement: Supplementary file 2 [file DataSheet1.ZIP › Raw Data 1/Figure 2/Drug accumulation in fluorescence microscopy/Mitoxantrone +Dorsomorphin 1μM S1-M1-80 sgABCG2.png]

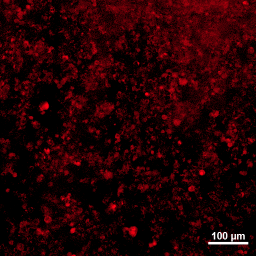

Supplement: Supplementary file 2 [file DataSheet1.ZIP › Raw Data 1/Figure 2/Drug accumulation in fluorescence microscopy/Mitoxantrone +Dorsomorphin 1μM S1-M1-80 Vector.png]

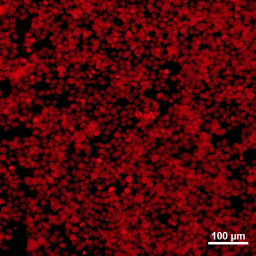

Supplement: Supplementary file 2 [file DataSheet1.ZIP › Raw Data 1/Figure 2/Drug accumulation in fluorescence microscopy/Mitoxantrone S1-M1-80 sgABCG2.png]

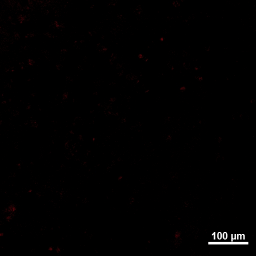

Supplement: Supplementary file 2 [file DataSheet1.ZIP › Raw Data 1/Figure 2/Drug accumulation in fluorescence microscopy/Mitoxantrone S1-M1-80 Vector.png]

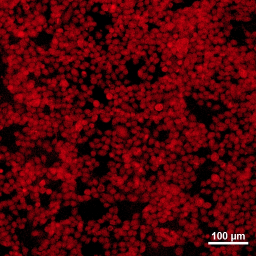

Supplement: Supplementary file 2 [file DataSheet1.ZIP › Raw Data 1/Figure 2/Drug accumulation in fluorescence microscopy/Mitoxantrone+KU55933 1μM S1-M1-80 sgABCG2.png]

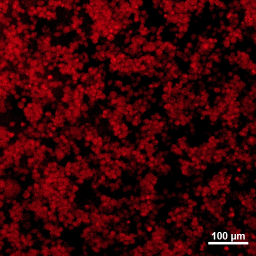

Supplement: Supplementary file 2 [file DataSheet1.ZIP › Raw Data 1/Figure 2/Drug accumulation in fluorescence microscopy/Mitoxantrone+KU55933 1μM S1-M1-80 Vector.png]

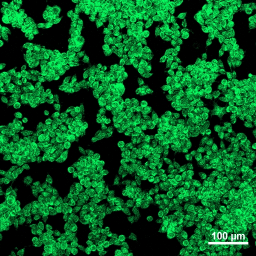

Supplement: Supplementary file 2 [file DataSheet1.ZIP › Raw Data 1/Figure 2/Drug accumulation in fluorescence microscopy/Rhodamine123 +Dorsomorphin 0.3μM S1-M1-80 sgABCG2.png]

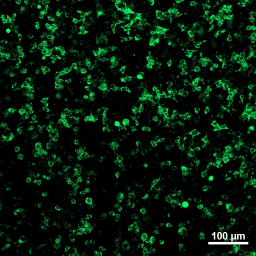

Supplement: Supplementary file 2 [file DataSheet1.ZIP › Raw Data 1/Figure 2/Drug accumulation in fluorescence microscopy/Rhodamine123 +Dorsomorphin 0.3μM S1-M1-80 Vector.png]

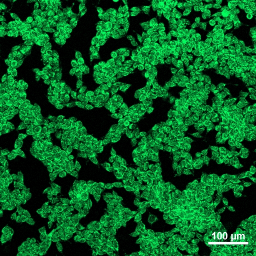

Supplement: Supplementary file 2 [file DataSheet1.ZIP › Raw Data 1/Figure 2/Drug accumulation in fluorescence microscopy/Rhodamine123 +Dorsomorphin 1μM S1-M1-80 sgABCG2.png]

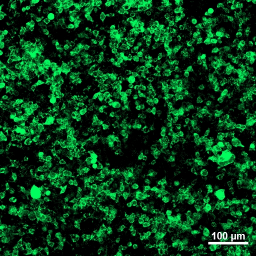

Supplement: Supplementary file 2 [file DataSheet1.ZIP › Raw Data 1/Figure 2/Drug accumulation in fluorescence microscopy/Rhodamine123 +Dorsomorphin 1μM S1-M1-80 Vector.png]

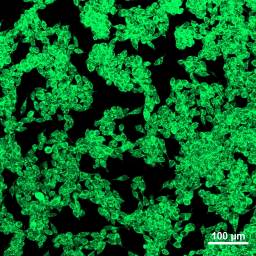

Supplement: Supplementary file 2 [file DataSheet1.ZIP › Raw Data 1/Figure 2/Drug accumulation in fluorescence microscopy/Rhodamine123 +KU55933 1μM S1-M1-80 sgABCG2.png]

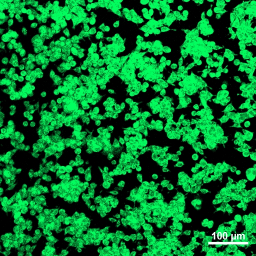

Supplement: Supplementary file 2 [file DataSheet1.ZIP › Raw Data 1/Figure 2/Drug accumulation in fluorescence microscopy/Rhodamine123 +KU55933 1μM S1-M1-80 Vector.png]

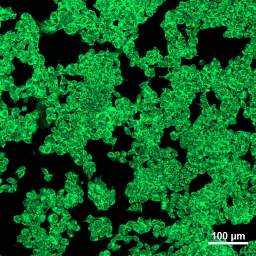

Supplement: Supplementary file 2 [file DataSheet1.ZIP › Raw Data 1/Figure 2/Drug accumulation in fluorescence microscopy/Rhodamine123 S1-M1-80 sgABCG2.png]

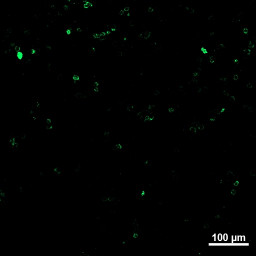

Supplement: Supplementary file 2 [file DataSheet1.ZIP › Raw Data 1/Figure 2/Drug accumulation in fluorescence microscopy/Rhodamine123 S1-M1-80 Vector.png]

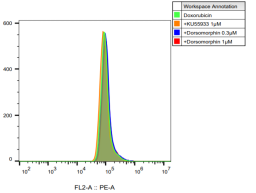

Supplement: Supplementary file 2 [file DataSheet1.ZIP › Raw Data 1/Figure 2/S1-M1-80 sgABCG2 Dox.png]

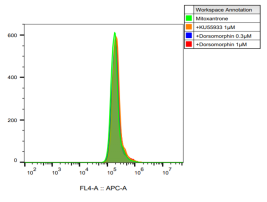

Supplement: Supplementary file 2 [file DataSheet1.ZIP › Raw Data 1/Figure 2/S1-M1-80 sgABCG2 Mito.png]

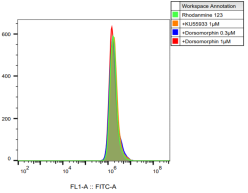

Supplement: Supplementary file 2 [file DataSheet1.ZIP › Raw Data 1/Figure 2/S1-M1-80 sgABCG2 Rho.png]

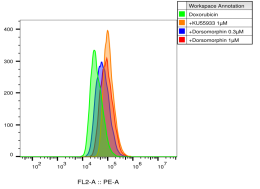

Supplement: Supplementary file 2 [file DataSheet1.ZIP › Raw Data 1/Figure 2/S1-M1-80 Vector Dox.png]

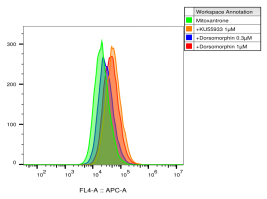

Supplement: Supplementary file 2 [file DataSheet1.ZIP › Raw Data 1/Figure 2/S1-M1-80 Vector Mito.png]

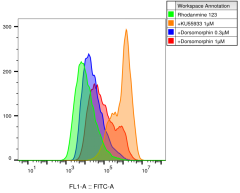

Supplement: Supplementary file 2 [file DataSheet1.ZIP › Raw Data 1/Figure 2/S1-M1-80 Vector Rho.png]

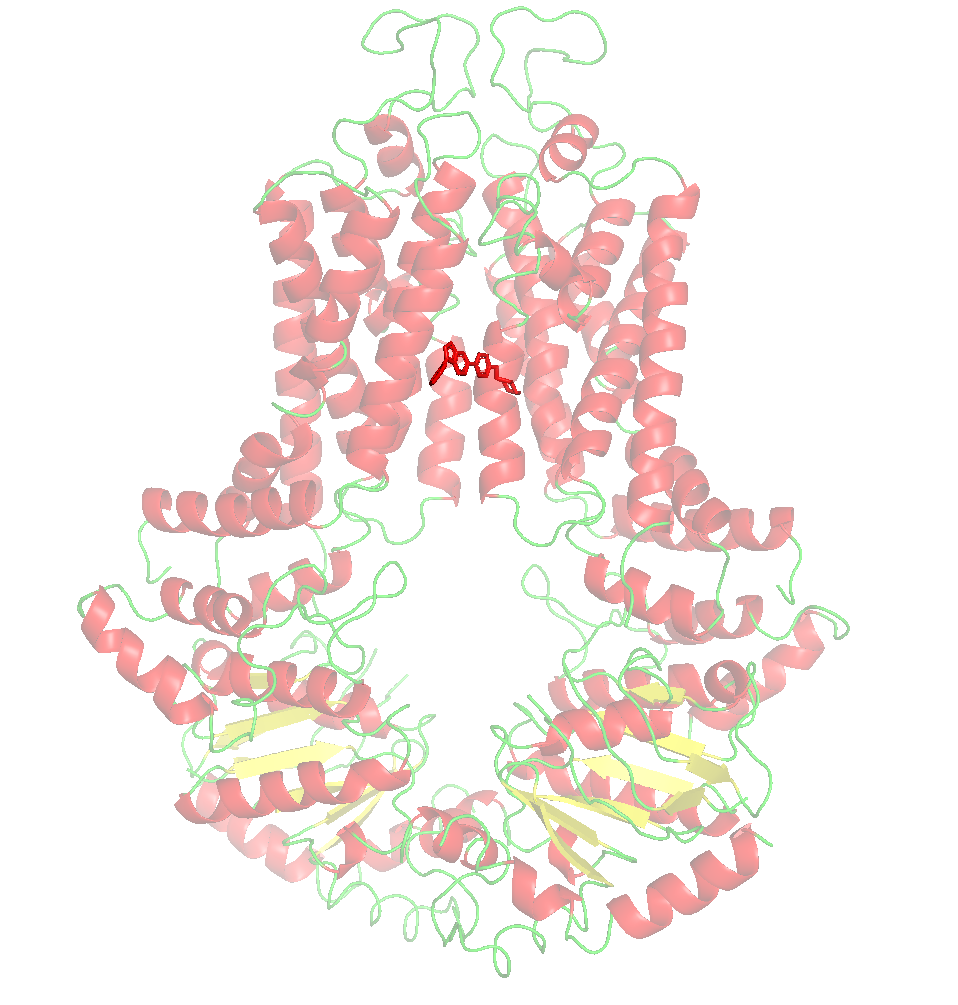

Supplement: Supplementary file 2 [file DataSheet1.ZIP › Raw Data 1/Figure 3/Molecular docking1.png]

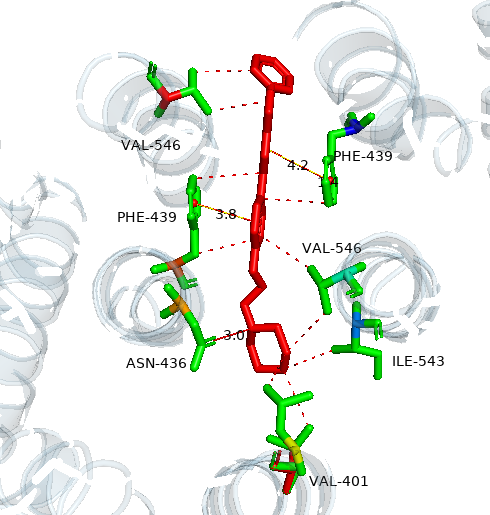

Supplement: Supplementary file 2 [file DataSheet1.ZIP › Raw Data 1/Figure 3/Molecular docking2.png]

## Slide 1
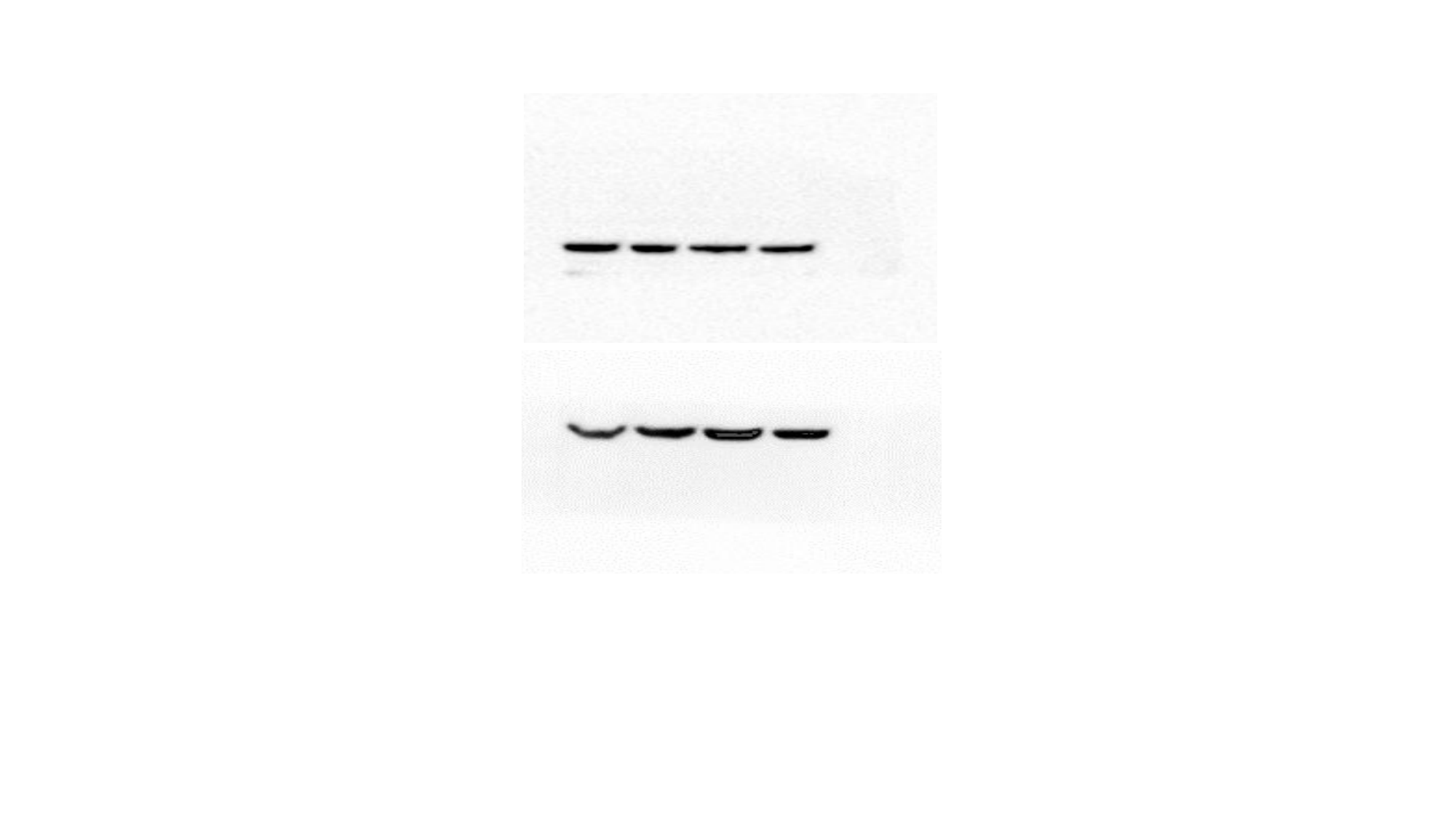

Supplement: Supplementary file 2 [file DataSheet1.ZIP › Raw Data 1/Figure 3/Original Images for Blots.pptx]
